# *Toxoplasma gondii* Recombinant Antigens in the Serodiagnosis of Toxoplasmosis in Domestic and Farm Animals

**DOI:** 10.3390/ani10081245

**Published:** 2020-07-22

**Authors:** Bartłomiej Ferra, Lucyna Holec-Gąsior, Weronika Grąźlewska

**Affiliations:** Department of Molecular Biotechnology and Microbiology, Faculty of Chemistry, Gdansk University of Technology, Narutowicza 11/12 Str., 80-233 Gdansk, Poland; lucholec@pg.edu.pl (L.H.-G.); wergrazl@student.pg.edu.pl (W.G.)

**Keywords:** *Toxoplasma gondii*, toxoplasmosis, recombinant antigens, serodiagnosis, animals

## Abstract

**Simple Summary:**

The very common parasite infections in animals are caused by members of Apicomplexa, including *Toxoplasma gondii*, *Neospora* sp., and *Sarcocystis* sp. These parasites pose serious veterinary problems. For example, the development of unambiguous diagnostic algorithms and determining the correct diagnosis are hindered by the similar antigenic structure of these parasites, as well as the multitude of similar disease symptoms presented in an infected animal. The intracellular parasite, *T. gondii*, infects a wide range of warm-blooded animals, including humans. This parasite is widespread among different animal populations, contributes to the loss of reproductive and malformations in young individuals, and can become a serious economic concern for farmers. Additionally, the consumption of undercooked or raw meat and the consumption of improperly processed milk product derived from farm animals are the main parasite transmission routes in humans. This work reviews potential improvements to diagnostic techniques that use recombinant antigens for serodiagnosis of toxoplasmosis in various species of animals.

**Abstract:**

Toxoplasmosis is caused by an intracellular protozoan, *Toxoplasma gondii*, and is a parasitic disease that occurs in all warm-blooded animals, including humans. Toxoplasmosis is one of the most common parasitic diseases of animals and results in reproductive losses. Toxoplasmosis in humans is usually caused by eating raw or undercooked meat or consuming dairy products containing the parasite. Diagnosis of toxoplasmosis is currently based on serological assays using native antigens to detect specific anti-*T. gondii* antibodies. Due to the high price, the available commercial agglutination assays are not suited to test a large number of animal serum samples. The recent development of proteomics elucidated the antigenic structure of *T. gondii* and enabled the development of various recombinant antigens that can be used in new, cheaper, and more effective diagnostic tools. Continuous development of scientific disciplines, such as molecular biology and genetic engineering, allows for the production of new recombinant antigens and provides the basis for new diagnostic tests for the detection of anti-*T. gondii* antibodies in animal serum samples.

## 1. Introduction

Toxoplasmosis is a widespread parasitic infestation caused by the ubiquitous, obligate, intracellular parasite, *T. gondii*, that infects a wide range of hosts, including humans [1]. Toxoplasmosis poses a serious problem in veterinary medicine because the parasite infects a wide range of warm-blooded animals and is considered one of the main causes of reproductive losses in farm-raised sheep, goats, cows, and pigs. The risk of toxoplasmosis in animals is similar to that in humans, and the risk is dependent on the type of farm management, the animal diet, and the geographical region, as well as of farms’ sanitary conditions. Among farmed animals*, T. gondii* tissue cysts are most often detected in sheep, goats, cows, and pigs, and less frequently in chickens, rabbits, and horses [1]. The most common source of animal infections is sporulated oocysts that enter the animals, either through food or water. Hay, straw, and grain, both a source of food and/or litter, often become contaminated with cat faeces containing oocysts and are a primarily source of infection for many farm animals. *T. gondii* infection in farm animals causes abortion and stillbirth, and it results in not only significant reproductive losses but significant economic losses, as well. Offspring from animals infected with *T. gondii* during the later stages of pregnancy are generally weaker. These young often die within a few weeks after birth. Some adult animals with congenital toxoplasmosis may even become barren [2]. Under natural conditions, seroprevalence of toxoplasmosis in farm animals depends on the age of animals, the geographical area, the hygienic condition of the farms, and farm management, among others. Based on data obtained in some regions of Europe, seroprevalence of toxoplasmosis may be varied, from 4% up to 92% in sheep (average 35.9%) [1,3], from 0% up to 80% in horses (average 25.8%) [4], and from 0% up to 64% in pigs (average 6%) [1,3].

The World Health Organization (WHO) estimates that one-third of the human population is infected with *T. gondii*. According to the WHO reports, over 60% of population has been infected with *T. gondii* in some regions of the world. The incidence of infection depends on the geographical region, diet and sanitation. People become infected with a parasite, most commonly by the oral route (over 80% of cases), through the consumption of raw or undercooked meat containing tissue cysts, or vegetables and fruits contaminated with the soil containing oocysts shed by cats [1,5,6].

## 2. *Toxoplasma gondii* Antigenic Structure—Antigens and Antigen Complexes

For several decades, research has been conducted to determine the full antigenic structure of *T. gondii*. Based on the results obtained to date, antigens involved in the individual stages of parasite invasion into the host cells has been elucidated. This knowledge informs the development of: (1) drugs that block cellular mechanisms essential for the initial stages of parasitic invasion; and (2) vaccines that stimulate the production of specific antibodies directed against parasite surface antigens. Knowledge of the parasite’s antigenic structure is also important from the diagnostic point of view, as it furthers the development of new diagnostic tools. *T. gondii* antigens are found on the surface of the parasite’s cell membrane and in the cytosol, as well as, in secretory organelles, such as rhoptry, micronemes, and high-density granules released during the invasion of the parasite into the host cell. *T. gondii* antigens also fill the interior of a parasitophorous vacuole and tissue cyst. Four main groups of *T. gondii* antigens have been identified and are discussed below [7,8,9,10,11,12,13].

### 2.1. Surface Antigens (SAG)

The surface of *T. gondii* is coated with a family of developmentally regulated glycosylphosphatidylinositol (GPI)-linked proteins, SAG1-related sequence (SRS) protein families, that are the most well-known tachyzoite proteins studied to date. SRS proteins mediate attachment to host cells and interface with the host immune response to regulate the virulence of the parasite; the most well-studied of all SRS proteins is SAG1 [10,14,15,16,17,18]. Some surface antigens are typical for a certain parasite’s stage, e.g., the SAG1 antigen, as well as SAG2A/B [10,17], SRS3 [16], and SAG3 [19], are expressed on the surface of tachyzoites [14], whereas SAG2D [17] and BSR4 [16] are bradyzoite-specific proteins.

### 2.2. Microneme Antigens (MIC)

The MIC antigens are a group of antigens whose production depends on the intracellular concentration of calcium ions. MIC antigens form adhesive complexes or occur in the form of single proteins and are secreted from specialised secretory organelles called micronemes. MIC antigen functions include adhesion and disturbance of the integrity of the host cell membrane to facilitate the parasite penetration inside the host cells. MICs possess adhesive motifs usually found in higher eukaryote proteins [20] and have a functional role in host cell attachment, motility, invasion, and have a synergistic role in the infectious process [21,22,23,24,25,26,27]. Three distinct microneme protein complexes have been identified to date, MIC6-MIC1-MIC4, MIC8-MIC3, and MIC2-M2AP. The MIC6-MIC1-MIC4 and MIC8-MIC3 complexes are responsible for targeted adhesion to surface receptors of host cells, creating a connection between the parasite and the host cell [22,27]. Microneme proteins that build the MIC2-M2AP complex also play a fundamental role in movement and penetration of the parasite into host cells. Another important antigen is AMA1, which, together with rhoptry neck (RON) proteins, forms a mobile connection, so-called “moving junction” (MJ) complex [28].

### 2.3. Rhoptry Neck (RON) and Rhoptry Antigens (ROP)

Rhoptry neck (RON) and rhoptry antigens (ROP) are a group of proteins secreted from the neck or bulb of the rhoptries that facilitate coronal folding of the host cell and formation of parasitophorous vacuoles (PV). All ROPs contain a signal peptide and many have at least one predicted transmembrane domain or a GPI anchor, suggesting an association with membranes. In addition, rhoptry proteomic analysis has led to the characterisation of proteins specifically localised in the rhoptry neck, i.e., RONs. Many ROPs and RONs contain repeated motifs that may be involved in protein–protein interactions. Virtually all the information regarding the contents or functions of the rhoptries has come from the research on *T. gondii* tachyzoite stage [29,30]. Only one bradyzoite-specific ROP has been described [31]. ROP antigens are mainly associated with the formation of PV and parasitophorous vacuole membranes (PVM), whereas RON proteins mainly participate in the creation of the MJ complex; the most important complexes are AMA1-RON2-RON4/5/8 [32,33].

### 2.4. Dense Granules Antigens (GRA)

Antigens secreted by high-density granules are classified as excreted-secreted antigens (ESA), and constitute up to 80% of antigens detected in the early phase of toxoplasmosis. The GRA antigens are characterised by a relatively low molecular weight of proteins ranging from 21–41 kDa [19,34]. All GRA proteins secreted by high-density granule vesicles play an important role in structural modifications of the PV, particularly in the construction of the internal membranous nanotubular network (MNN) microtubules and PVM, where *T. gondii* parasites develop [8,35,36,37]. Antigens from the GRA group are a protein family with an unusual structure. Along with the development of techniques used in molecular biology, reports have recently proposed the structures and functions of specific antigens belonging to this family.

## 3. Animal Toxoplasmosis—Prevalence, Symptoms, and Diagnosis

In many cases, the protozoan *T. gondii* infection is asymptomatic in animals, and the only confirmation of infection is the presence of specific anti-*T. gondii* antibodies. If symptoms associated with toxoplasmosis occur, they are most often non-specific, e.g., lack of appetite, diarrhoea, and vomiting, as well as significant weight loss and weakness. The most serious consequences of infection are observed in sheep, goats, pigs, and cows. In these animals, toxoplasmosis may have serious clinical consequences associated with reproductive potential. Similar to humans, the parasite can be transmitted from mother to foetus and often leads to abortions. In early pregnancy, ingestion of oocysts and the development of primary infection usually results in the death of the embryo, its decomposition, and absorption within the uterus. In this phase of pregnancy, miscarriages and foetal mummification may occur. Animals infected in the later stages of pregnancy may have weaker offspring, and young ones may survive, only to die within a few weeks after delivery. In addition, young may be unable to feed themselves. Some adult animals may become unable to reproduce if infected during pregnancy [2].

Cats are the ultimate hosts of the *T. gondii* parasite and are an interesting group of animals to screen for infections. The percentage of infected cats around the world varies depending on the age and environment. In the United States, the largest diagnostic study for this group of animals found the presence of specific antibodies to *T. gondii* in 31% of the population studied [38]. Compared to European countries, the percentage of seropositive cats in the U.S. is lower than recorded in France (43%), Sweden (42%), or Turkey (43%). Comparable results were obtained in Italy (9–33%), while the highest was recorded in the Czech Republic (59%), Slovenia (57%), and in Germany (34–51%) [39]. It should be noted that the detection of parasite infection in cats may not play a significant role in preventing the spread of oocysts excreted by cats into the environment. In most cases the primary infection with *T. gondii* in cats does not cause immunosuppression [40,41,42]. The specific immunoglobulin G (IgG), immunoglobulin M (IgM), and immunoglobulin A (IgA) antibodies appear in the serum of cats when they show clinical signs of disease and are secreting oocysts [43]. Diagnosis of toxoplasmosis in cats, therefore, may be most relevant to the selection of cats that are candidates for vaccination, as evidenced by the oral vaccination of healthy cats with bradyzoites of the mutated strain *T. gondii* T-263 that led to an intestinal parasite infection but did not cause oocyst production [44,45]. In addition, one long-term study showed that, several years after vaccination of cats on farms, parasite infections were not found in caught wild mice, and there was a significant decrease in the percentage of seropositive pigs on these farms [46]. Vaccination of cats with *T. gondii* mutant strain T-263 was associated with the benefit of a very low likelihood in cyst formation compared to natural infected cats [47,48]. Although primary infection is usually asymptomatic in cats, in the long term, it is often associated with adverse effects within the abdominal cavity and lungs. Histopathological examination of the abdominal cavity in cats with toxoplasmosis revealed, among other conditions, foci of necrotic hepatitis, as well as congestion and oedema of many internal organs [49].

Of the animals in which meat is consumed by humans, sheep are most susceptible to infection with protozoa. Infection with *T. gondii* is a serious problem in sheep breeding, causing a significant reduction in the reproductive capacity of these animals. Studies from Serbia show that animals bred in large herds reported more seropositive results compared to animals from small farms [50]. In Europe, the average incidence of sheep toxoplasmosis is 35.9% and varies from 4–92% in individual countries [1,3]. High susceptibility of sheep to parasitic infection is due to being raised mainly on pastures, where they can eat oocysts that have contaminated their food and water sources. Studies show that parasite infection is responsible for 25% of all abortion cases found in sheep and in extreme cases may lead to the inability of these animals to reproduce [2,51]. Unless primary infection occurred during pregnancy, sheep generally remain resistant to re-infection; thus, the risk of producing contagious offspring in subsequent pregnancies is negligible. When primary infection occurs during pregnancy, the probability of tachyzoite crossing the placenta into the foetus is greater than 80% and, depending on the time of infection, may lead to resorption, abortion, delivery of dead lambs, or delivery of lambs with many malformations [2]. *T. gondii* cysts can be found in up to 30% of mutton that is sold, so sheep meat can be a potential source of transmission of the parasite to humans [6]. Diagnosis of toxoplasmosis in sheep is crucial, not only because of the potential elimination of the source of transmission of the parasite but also to aid in the selection of healthy animals that can be vaccinated with the Ovilis™ Toxovax vaccine that is approved in some countries.

Sheep farming illustrates the economic problems of parasitic infections; for example, wool from sheep infected with protozoa has significantly decreased quality compared to the wool of healthy animals. Toxoplasmosis is the cause of other economic losses for sheep breeders due to spontaneous abortion in sick, pregnant females. For example, in 2003, 86 million sheep were raised in 15 European Union countries, and studies showed toxoplasmosis was responsible for 1–2% of deaths in newborn lambs per year, and a loss of over 1.25 million sheep of breeding age per year [50].

In many countries, pig meat is one of the most consumed by humans, therefore; pork containing tissue cysts from the parasite can be a potential source of human infection. The average seroprevalence of toxoplasmosis in pigs is estimated to be between 10–20% [52]. However, the incidence of toxoplasmosis in pigs throughout European countries ranges from 0–64% [1,3]. While the clinical view of primary infection in pigs is like that of sheep, abortion in pregnant sows is much rarer and is probably associated with a significantly lower risk of the parasite passing through the placenta to the foetus [52]. Parasitic infection is most common in young pigs, like sheep, by the ingestion of contaminated food and water. For pigs, the incidence of infection depends primarily on the type of farm; the research shows that a very high percentage of seropositive pigs (up to 100%) are on free-range farms. For pigs raised on closed farms, where strict sanitary conditions are respected regarding both living conditions and the quality of feed and water fed to animals, the risk of infection can be eliminated [1,53]. While closed animal husbandry allows you to minimise, and sometimes even eliminate, the risk of parasite infection, European Union regulations call for phasing out closed farms in favour of free-range farming. The problem with this plan has recently been pointed out by Dubey et al. [54], who, for the first time in several years, recorded a high percentage of seropositive pigs in the United States in “organic” farms. Diagnosis of toxoplasmosis in pigs, therefore, will continue to be very important from the point of limiting the risk of transmission of the parasite to humans, especially pregnant women.

Consumption of horse meat constitutes an important part of the culinary traditions in the countries of Central Asia and South America, as well as in some European countries, such as France and Italy. Most often, fresh horse meat is consumed in the form of Japanese basashi, Italian carpaccio, or tartare. Therefore, eating raw horse meat is a potential source of parasite transmission for humans. In Europe, the average seroprevalence of toxoplasmosis in horses has been estimated at 25.8%, but the incidence in individual countries ranges from 0–80% [4]. *T. gondii* infection has been reported in an increasing number of both terrestrial and marine animal species. Among land animals, *T. gondii* infection was detected not only in farm animals (e.g., pigs, sheep, goats, chickens, horses, and cows) but also in wild game (e.g., roe deer, wild boar, deer, hares), fur animals, and many exotic animals (e.g., kangaroos, camels, yaks) [1,39,55,56,57]. *T. gondii* infection poses a threat to marine mammals, such as the sea otter, Hawaiian monk seal, and beluga whale [58,59,60]. Moreover, the presence of *T. gondii* has been found in marine fish [61,62], as well as in arachnids and, more precisely, in ticks [63].

Although the epidemiology of parasitic diseases in farmed animals should be monitored in the European Union following Directive 2003/99/EC, in many countries there are no legal regulations that would impose control over the incidence of *T. gondii* infection [64]. Diagnosis of toxoplasmosis is primarily accomplished with indirect tests, based on serological tests that detect antibodies of particular immunoglobulin classes. Such tests should be characterised by high sensitivity and specificity, obtaining reproducible results, and simplicity of performance. The first test used in the diagnosis of toxoplasmosis was the dye test (DT) developed by Sabin and Feldman [65]. Despite the DT’s high specificity and sensitivity, it is currently only used as a reference test due to the limitation of being unable to differentiate immunoglobulin classes. Currently, toxoplasmosis diagnostics primarily use enzyme-linked tests, the most common of which is enzyme-linked immunosorbent assay (ELISA). The most popular tests for the detection of toxoplasmosis in animals are summarised in Table 1.

## 4. Recombinant Antigens in the Diagnosis of Toxoplasmosis

The diagnosis of toxoplasmosis is based primarily on serological tests. The sensitivity and specificity of these tests depend on several factors, the most important of which is the level of individual classes of antibodies produced during infection and the quality of the antigens used in the tests. In principle, a person can only affect the quality and purity of antigens. Native antigens (for example, *Toxoplasma* Lysate Antigen (TLA)) are the most common in the diagnosis of toxoplasmosis. Unfortunately, the production of these antigens is expensive, time-consuming, and labour-intensive, and is associated with the danger of the diagnostician becoming infected with a parasite due to the necessity to grow *T. gondii* in the laboratory. Therefore, many laboratories are working on the production of recombinant antigens in well-characterised expression systems (e.g., *Escherichia coli*). Modern genetic engineering tools allow both clone and the subsequent expression scheme fusion proteins with additional domains at the N- and C-terminus to be developed, thus enabling subsequent effective purification to obtain high-purity protein preparations. The undisputed advantage of this approach in antigen production is the ability to easily optimise expression for high yield. In addition,, the risk of infection resulting from working with pathogenic microorganisms is eliminated. This makes it possible to obtain individual antigens easily and quickly. These antigens can be used as selectable markers to determine individual phases of the disease. The advantage of producing recombinant *T. gondii* antigens is the ability to use single proteins or their mixtures to determine the phase of the disease, which is particularly important in determining a diagnosis and treatment. Use of recombinant proteins in tests results in repeatability of results, easier standardisation, and consistency of protein preparation.

The prevalence of toxoplasmosis among animals has a significant impact on the risk of this disease infecting humans. Felids, the ultimate hosts of the parasite, are a potential source of parasite infection for other animals and humans. The meat of infected farmed animals is an important source for parasite transmission to humans. Currently, various types of agglutination tests are used to diagnose animal toxoplasmosis, which is expensive, and therefore, not suitable for testing large animal populations. Over the past 30 years, the diagnostic utilities of many recombinant *T. gondii* antigens have been evaluated for the detection of specific antibodies in human serum samples [66,67].

Some research teams also confirm the utility of recombinant antigens to detect specific anti-*T. gondii* antibodies in animal sera. All recombinant antigens tested in animals, to date, have been obtained in the *E. coli* prokaryotic expression system. This approach quickly produces significant amounts of protein that can be easily purified from cell lysate using one-step metal affinity chromatography due to the presence of polyhistidine domains in the protein. The diagnostic utility of recombinant antigens has generally been determined in IgG ELISAs or simple immunochromatographic tests (ICT) based on single proteins, mixtures thereof, and recombinant chimeric antigens, which formed by combining two or more immunodominant fragments of different parasite proteins. Widespread use of IgG ELISA tests for research is due to the following facts: (a) high sensitivity and specificity of the test, (b) most laboratories have the necessary equipment, (c) the ability to test many samples at the same time, (d) possibility of performing several repetitions in one test, (e) the ability to perform tests for several antigens simultaneously, (f) possibility of performing reference tests based on native antigen, e.g., TLA, and (g) the ability to easily and quickly compare test results based on different antigenic preparations. All tests performed to date have been based on the detection of IgG antibodies, due to market needs; commercially available tests dedicated to testing animal sera detect only IgG antibodies. From a veterinary perspective, it is important to simply determine whether the animal is infected with a parasite or not infected. In this case, determining the phase of the disease is not as important as it is in humans. Moreover, it is unlikely that any animal breeder would decide to regularly examine entire herds of animals due to the costs that would be incurred and lack of regulatory requirements.

The results of the review of diagnostic utility of recombinant antigens collected are shown in Table 2 (feline sera), Table 3 (canine sera), Table 4 (ovine sera), Table 5 (bovine sera), Table 6 (caprine sera), Table 7 (porcine sera), Table 8 (equine sera), Table 9 (chicken sera), and Table 10 (donkey sera). Tables contain information, such as: recombinant antigen used in the study, expression system in which the antigen was obtained, test in which the diagnostic usefulness of the antigens was tested, performed reference test, and obtained results (sensitivity, specificity, and comparison to reference test results).

## 5. Discussion

*T. gondii* infections are widespread among both wild and domestic animals. This parasitic infection is a significant problem in farmed animals and is associated with serious reproductive impairments and economic losses. In addition, transmission of the parasite to humans most often occurs through the consumption of raw or undercooked meat and unpasteurised dairy products, as well as water, fruit, or vegetables contaminated with oocysts. In pregnant women, 30–63% of *T. gondii* infections are caused by eating inadequately prepared meat [104]. Vegetables and fruits potentially contaminated with oocysts can be washed before consumption. Meat, however, may contain parasite cysts in the tissue and the consumer is not always aware of the risk of infection. Admittedly, proper heat treatment, curing, and earlier freezing of meat causes the deactivation of cysts; however, in the culture of many countries, culinary traditions include dishes made from fresh, raw meat. Most consumers, therefore, often eat undercooked meat without being aware of the risk of infection and has been confirmed by studies in which the incidence of parasite infection in meat eaters was compared to vegetarians. Consumption of undercooked or cured meat products it can cause from 30% to 63% of cases of *T. gondii* infection, while contact with products from the soil (fruit and vegetables) from 6% to 17% [104,105]. In the absence of a vaccine that could be used universally in all animals and humans, diagnostic tools that would allow the rapid and cheap testing of numerous samples of biological material from farm animals are being developed. Some researchers suggest tests for processed meat should be required. For example, Kijlstra and Jongert [64,106] suggest that meat with negative tests should be labelled free from *T. gondii*, and meat with positive tests be subjected to thermal pre-treatment or freezing and then sold as meat safe from *T. gondii.* Another alternative may be to provide information to the consumer on the type of management used to produce and process the meat, as well as if it was carried out following accepted standards under strict veterinary control, and random testing protocols [104].

This paper presents a review of the potential utility of recombinant antigens for serodiagnosis of toxoplasmosis in various animal species. Unfortunately, toxoplasmosis diagnostics based on recombinant antigens is not as developed for animals as it is for humans. Most recombinant antigens investigated for diagnostics method in animals have been limited to trials in cats, sheep, and pigs. After analysing research results from around the world, one can conclude that there is a paucity of information even though the need for development of rapid, reliable diagnostics is well demonstrated. First, there is the lack of research on cross-reactions with other Apicomplexa type parasites. Only a few publications raised the issue of cross-reactions between *T. gondii* and *N. caninum*, which are the most antigenically similar among Apicomplexa parasites. In even fewer works, *N. caninum* seropositive sera were used to determine cut-off values or as controls [80,84,85,89,91]. Apicomplexa-type parasites are antigenically similar and additional tests on sera from animals infected with other parasites must be performed to fully assess the diagnostic potential of recombinant antigens, in particular, the species *Neospora* sp. and *Sarcocystis* sp. The problem of cross-reactions may be particularly important in the case of animal species that are definitely more susceptible to infection with *N. caninum* and *N. hughesi* than to infection with *T. gondii* [107,108].

Animal sera are generally tested with commercially available latex agglutination test (LAT), modified agglutination test (MAT), immunofluorescence antibody test (IFAT), or Western blotting tests based on native antigens. It is well known that the sensitivity and specificity of these tests are different but often not reported in the studies. Sometimes the variation made it difficult to classify the tests as positive and negative based on recombinant antigens. Particularly, published research on the recombinant GRA7 antigen is sparse and should be further investigated. It cannot be assumed that the recombinant GRA7 protein, tested in feline sera and developed for the IgG ELISA test with 89.7% sensitivity and 92.5% specificity [75], will be equally good for detecting anti-*T. gondii* antibodies in ovine or bovine species [88,90]. This concern is especially important to consider when using serum samples from naturally infected animals that have not been tested by any commercial test or even an in-house ELISA test based on native antigens (e.g., TLA) to provide comparison and standardisation controls. A good rule is to test sera with at least two commercial tests (e.g., MAT and IFAT) and then compare the results obtained with the recombinant antigens to the results from commercial tests. Using this rule, it will be possible to fully understand the diagnostic potential of recombinant antigens for detecting anti-*T. gondii* antibodies in animal sera. To obtain the best results, studies show that the development of new diagnostic tests requires the use of mixtures of recombinant antigens or recombinant chimeric antigens that consist of two or more immunodominant fragments of different proteins. Nevertheless, it is possible recombinant antigen may be recognised by anti-*T. gondii* antibodies found in the sera of three animal species, but antibodies from another two animal species may not detect the antigen. This type of result can be expected because animal species have developed diverse resistance mechanisms to parasite infection; for example, humans and sheep are more susceptible to parasite infection than horses or cattle [109]. The evolution of individual mammalian species has resulted in different changes in genomes, silencing some genes and removing others. An example that perfectly illustrates this fact is the variable number of *irg*-related genes that encode proteins from the GTPase family associated with innate immunity against intracellular pathogens. Genes encoding IRG proteins are found in virtually all mammals, and a total of 21 genes encoding proteins from the IRG family can be found in the mouse genome; however, in the human genome, they have been reduced to 2 genes, namely *irgm* and *irgc*, with the second gene probably not coding for the protein associated with immunity [110,111,112,113,114,115,116,117,118]. Additionally, varied resistance to infection occurs between species strains sometimes differ in individual genes, and is perfectly illustrated by the animal model most commonly used for experiments, namely different strains of laboratory mice [119,120,121]. Studies of inbred mice of the BALB/c strain show relatively less sensitivity to *T. gondii* infection; the C3H/HeJ strain is moderately sensitive, and the C57BL/6 strain sensitive to infection. Of course, this does not mean that BALB/c mice will not become infected with a parasite. On the contrary, it is an excellent animal model used for research on the acute phase of the disease. The aforementioned strains of mice are primarily characterised by a different survival capacity, depending on the strain and developmental form of the parasite used to infect them, which suggests animal species will have different immune responses due to infection with a given *T. gondii* strain. The virulence of individual strains of the parasite may, therefore, determine the modulation of the immune response in various animal species, and this may also affect the titre of specific antibodies directed against individual parasite proteins in the serum. Furthermore, it can be assumed that individual animal species may be more or less susceptible to infection with a given *T. gondii* strain. 

This work presents an overview of the potential utility of recombinant antigens for serodiagnosis of toxoplasmosis in various species of animals. Research to date has shown the potential diagnostic utility of *T. gondii* recombinant antigens for the detection of parasitic infection in some animal species. This review demonstrates the difficulties that may be encountered when developing a unique diagnostic algorithm that can be universally used for all animal species. However, the future developments of molecular biology, genetic engineering, and immunology techniques may allow for the development of a universal species-independent test that allows detection of anti-*T. gondii* antibodies. Future research will determine if this will be possible, but remember that, until the proteome of all Apicomplexa-type parasites is known, possible cross-reactions may not be eliminated, which are the basic problem in differentiating these parasites in diagnostic methods today.

## 6. Conclusions

The prevalence of toxoplasmosis among animals is a significant diagnostic problem, especially for research carried out in recent years that concerned the detection of parasitic infection in a variety of animal species, including aquatic mammals. Epidemiological studies in animals is often carried out due to purely economic reasons, an excellent example of which is sheep infected with a parasite associated with serious reproductive losses. On the other hand, monitoring epidemiological situations can also be dictated by appropriate legal regulations. For example, European Union Directive No. 2003/99/EC imposes on the European Union countries the obligation to introduce programs enabling the determination of the prevalence of parasitic diseases, especially among farmed animals (sheep, goats, pigs) [3,64]. Unfortunately, in most countries, the regulatory requirements are not used to form the basis of a surveillance and monitoring program that would determine the true economic and resource impact of the disease. The available data do not usually refer to the actual epidemiological situation of specific parasitic diseases throughout the country but most often relate to specific regions in which a centre performed research for purely scientific reasons. From a public health point of view, the diagnosis of toxoplasmosis in farmed animals and implementations of appropriate management protocols appears to be key to eliminating the main source of transmission of the parasite to humans, which is the consumption of meat from infected animals. Therefore, the development of new diagnostic tools in the form of recombinant antigens may be able to solve this problem soon. Once the diagnostic potential of recombinant antigens is fully understood, recombinant antigen may prove to be a cheaper and better alternative to native antigens and form the basis for development of new diagnostic algorithms.

## Figures and Tables

**Table 1:** Characteristics of tests used in serodiagnosis of toxoplasmosis in animals.

Method	Advantages	Disadvantages
Western blotting (WB)	Reference method Detection of IgG and IgM class antibodies Repeatability of results	Time-consuming
		Specialised equipment needed
		High-level of operator experience required
Immunofluorescence antibody test (IFAT)	Reference method	Microscope required Visual reading of results Possibility of cross-reactions Test based on *T. gondii* tachyzoites
	Detection of specific antibodies	
	The ability to determine antibody titres	
	Detection of antibody classes	
	Repeatability of results	
Direct agglutination test (DAT)	High sensitivity and specificity Detection of IgG and IgM class antibodies Simplicity of implementation	High IgG titres may mask the presence of IgM
		2-mercaptoethanol needed to bind natural and IgM antibodies
		Test based on *T. gondii* tachyzoites
Latex agglutination test (LAT)	Simplicity of implementation	No possibility to differentiate classes of antibodies
Modified agglutination test (MAT)	High sensitivity and specificity Detection of IgG and IgM class antibodies Simplicity of implementation	High IgG titres may mask the presence of IgM
		2-mercaptoethanol needed to bind natural and IgM antibodies
		Test based on *T. gondii* tachyzoites
ELISA	Quantitative and qualitative determination of antibodies and antigens in biological material	Specialised equipment needed Quality of the test dependent on antigen preparation
	High sensitivity and specificity	
	Detection of all class antibodies	
	Automation	

**Table 2:** Recombinant antigens used in detection of anti*-T. gondii* antibodies in feline serum samples.

Antigen	Expression System	Assay	Reference Test	Results	Reference Country
H4; H11; M: H4 + H11	*E. coli*	IgG ELISA	In-house TEA-ELISA	32 serum samples were tested.	[68] Germany
				H4-ELISA: sensitivity 100%; specificity 100%.	
				H11-ELISA: sensitivity 50%; specificity 100%.	
				M-ELISA: sensitivity 100%; specificity 100%.	
				TEA-ELISA: sensitivity 100%; specificity 100%.	
H4; H11; M: H4 + H11	*E. coli*	IgG ELISA	In-house TEA-ELISA IFAT	306 serum samples were tested.	[69] Germany
				H4-ELISA: sensitivity 93%; specificity 100%.	
				H11-ELISA: sensitivity 64%; specificity 100%.	
				M-ELISA: sensitivity 95%; specificity 100%.	
				TEA-ELISA: sensitivity 98%, specificity 99%.	
				IFAT: sensitivity 94%; specificity 92%.	
SAG1	*E. coli*	IgG ELISA	LAT WB	193 serum samples were tested.	[70] Japan
				Using the commercial ELISA and LAT tests the serum samples were divided into positive (40) and negative (153).	
				SAG1-ELISA: positive results for 40 (20.7%) serum samples.	
				1 serum sample was positive in LAT, negative in WB and SAG1-ELISA.	
				1 serum sample was in LAT, positive in WB and SAG1-ELISA.	
SAG1; SAG2	*E. coli*	IgG ELISA	LAT ELISA	192 serum samples were tested.	[71] Japan
				Using the commercial LAT test the serum samples were divided into positive (39) and negative (153).	
				40 (20.8%) of serum samples were positive in SAG1-ELISA.	
				42 (21.9%) of serum samples were positive in SAG2-ELISA.	
				38 (19.8%) of serum samples were positive in both SAG1-ELISA and SAG2-ELISA.	
				39 (20.3%) of serum samples were positive in LAT.	
				41 (21.4%) serum samples were positive in both SAG2-ELISA and WB.	
SAG2	*E. coli* (E) *Baculovirus* (B)	IgG ELISA	LAT	187 serum samples were tested.	[72] Japan
				Using the commercial LAT test the serum samples were divided into positive (35) and negative (152).	
				53 (28.3%) of serum samples were positive in S-SAG2-ELISA.	
				36 (19.7%) of serum samples were positive in E-SAG2-ELISA (tested group of serum samples 183).	
				35 (18.7%) of serum samples were positive results in LAT.	
				The authors suggest that the B-SAG2 antigen is more reactive, but in view of the large discrepancy with the commercial LAT test, it is difficult to agree with this statement.	
SAG2	*E. coli*	IgG ELISA IgG ICT	LAT	179 serum samples were tested.	[73] Japan
				41 (22.9%) of serum samples were positive in SAG2-ICT.	
				33 (18.4%) of serum samples were positive in LAT, constituting 95.5% compliance with SAG2-ICT.	
				35 (19.6%) of serum samples were positive in SAG2-ELISA, constituting 96.1% compliance with SAG2-ICT.	
SAG1; SAG2; GRA6	*E. coli*	IgG RDT	ELISA	182 serum samples were tested. Preliminary tests showed that SAG2 and GRA6 recombinant proteins are characterised by low reactivity. SAG1-RDT: sensitivity 100%; specificity 99.2%. Commercial ELISA: specificity 91.2%; specificity 98.7%.	[74] Korea
GRA7	*E. coli*	IgG ELISA	MAT IFAT In-house TLA-ELISA	185 serum samples were tested. Using the commercial IFAT and MAT tests the serum samples were divided into positive (39) and negative (146). TLA-ELISA: sensitivity 84.6%; specificity 99.3%. GRA7-ELISA: sensitivity 89.7%; specificity 92.5%.	[75] China
M: SAG1 + SAG2	*E. coli*	DFICT	ELISA	97 serum samples were tested.	[76] China
				Using the commercial ID Screen^®^ Toxoplasmosis indirect multi-species ELISA test, the serum samples were divided into positive (28) and negative (68).	
				DFICT-M: sensitivity 92%, specificity 93.1%.	
SAG2, GRA2, GRA6; GRA7; GRA15; MIC10; M1: GRA6 + GRA7; M2: GRA2 + GRA7; M3: SAG2 + GRA7; M4: SAG2 + GRA6; M5: GRA2 + GRA6 + GRA7 + GRA15; M6: SAG2 + GRA2 + GRA6 + GRA7 + GRA15	*E. coli*	IgG ELISA	LAT In-house TLA-ELISA	419 serum samples were tested. Using the commercial LAT test the serum samples were divided into positive (73) and negative (346). TLA-ELISA: sensitivity 97.3%; specificity 93.6%. SAG2-ELISA: sensitivity 91.9%; specificity 88.1%. GRA2-ELISA: sensitivity 27.0%; specificity 96.5%. GRA6-ELISA: sensitivity 82.4%; specificity 88.7%. GRA7-ELISA: sensitivity 35.1%; specificity 89.9%. GRA15-ELISA: sensitivity 17.6%; specificity 86.4%. MIC10-ELISA: sensitivity 16.2%; specificity 85.8%. M1-ELISA: sensitivity 74.3%; specificity 89.0%. M2-ELISA: sensitivity 44.6%; specificity 89.3%. M3-ELISA: sensitivity 90.5%; specificity 85.5%. M4-ELISA: sensitivity 94.6%; specificity 89.6%. M5-ELISA: sensitivity 70.3%; specificity 86.1%. M6-ELISA: sensitivity 89.2%; specificity 95.4%.	[77] Egypt
GRA7	*E. coli*	IgG ICT IgG ELISA	LAT In-house TLA-ELISA	100 serum samples were tested.	[78] Japan
				Using the commercial LAT test the serum samples were divided into positive (76) and negative (24).	
				74 of serum samples were positive in GRA7-ELISA.	
				74 of serum samples were positive in GRA7-ICT.	
				71 of serum samples were positive in TLA RH-ELISA.	
				70 of serum samples were positive in TLA PLK-ELISA.	
				70 of serum samples were positive in TLA VEG-ELISA.	
				The authors suggest that two serum samples produced a false positive result in the LAT test., but their tests were based on the GRA7 protein are characterised by 100% sensitivity and specificity.	

Explanation of abbreviations: M—antigens mixture; TEA—traditional ELISA antigen; RDT—rapid diagnostic test; DFICT—dynamic flow immunochromatographic test; TLA-RH—TLA obtained from the RH stain of *T. gondii*; TLA-PLK—TLA obtained from the PLK strain of *T. gondii*; TLA-VEG—TLA obtained from the VEG strain of *T. gondii*.

**Table 3:** Recombinant antigens used in detection of anti*-T. gondii* antibodies in canine serum samples.

Antigen	Expression System	Assay	Reference Test	Results	Reference Country
GRA1; GRA7	*E. coli*	IgG ELISA	MAT IFAT In-house TLA-ELISA	259 serum samples were tested.	[79] China
				Using the commercial MAT and IFAT tests, the serum samples were divided into positive (44) and negative (215).	
				GRA1-ELISA: sensitivity 81%, specificity 95.4%.	
				GRA7-ELISA: sensitivity 91%, specificity 97.7%.	
				TLA-ELISA: sensitivity 88.1%, specificity 96.8%.	
M: SAG1 + SAG2	*E. coli*	DFICT	ELISA	241 serum samples were tested. Using the commercial ID Screen^®^ Toxoplasmosis indirect multi-species ELISA test. the serum samples were divided into positive (57) and negative (184). DFICT-M: sensitivity 96.2%, specificity 96.8%.	[76] China
SAG2	*E. coli*	IgG ELISA	-	187 serum samples were tested.	[80] Japan Turkey
				37 (24.1%) serum samples were positive for the presence of anti-*T. gondii* antibodies.	
				Serum samples were not tested by any commercial assay.	
MAG1	*E. coli*	IgG ELISA	WB In-house TLA-ELISA	93 serum samples were tested.	[81] China
				Using the Western blotting test with TLA, the serum samples were divided into positive (33) and negative (60).	
				MAG1-ELISA: sensitivity 93.9%, specificity 98.3%.	
				TLA-ELISA: sensitivity 87.8%, specificity 96.7%.	

**Table 4:** Recombinant antigens used in detection of anti*-T. gondii* antibodies in ovine serum samples.

Antigen	Expression System	Assay	Reference Test	Results	Reference Country
H4; H11; M: H4 + H11	*E. coli*	IgG ELISA	In-house TEA-ELISA	26 serum samples were tested.	[68] Germany
				H4-ELISA: sensitivity 79%; specificity 100%.	
				H11-ELISA: sensitivity 43%; specificity 100%.	
				M-ELISA: sensitivity 79%; specificity 100%.	
				TEA-ELISA: sensitivity100%; specificity 100%.	
H11	*E. coli*	IgG ELISA	In-house TEA-ELISA In-house P30-ELISA In-house SA-ELISA	92 serum samples were tested. H11-ELISA: sensitivity 34%; specificity 89%. P30-ELISA: sensitivity 96%; specificity 100%. TEA-ELISA: sensitivity 96%; specificity 100%.	[82] Switzerland
MAG1	*E. coli*	IgG ELISA	LAT	175 serum samples were tested.	[83] Japan
				29 (16.6%) serum samples were positive in LAT test.	
				42 (24%) serum samples were positive in MAG1-ELISA.	
				27 (15.4%) serum samples had the same results in LAT test and MAG1-ELISA.	
				No additional tests were performed to confirm the presence of anti-*T. gondii* antibodies, so it is difficult to determine the diagnostic utility of MAG1 protein.	
GRA1; GRA2ex2; GRA4; GRA5; GRA6; GRA9; SAG1; SAG2; SAG4; BSR4; ROP1; ROP9; MIC1ex2; MIC1ex34; MIC3; MAG1; BAG1; LDH1; LDH2; M1: GRA1 + ROP1; M2: GRA1 + SAG2; M3: SAG2 + ROP1; M4: GRA1 + SAG2 + ROP1	*E. coli*	IgG ELISA	IFAT MAT In-house TLA-ELISA	Preliminary evaluation of single recombinant antigens in IgG ELISA with 2 positive and 2 negative serum samples. 8 antigens (GRA1, GRA9, SAG1, SAG2, SAG4, MIC1ex2, MIC3, and ROP1) were selected for further analysis with pool of 108 serum samples. 3 antigens (GRA1, SAG2, ROP1) were selected with the sensitivity (98.9–100%) and specificity (100%). Mixtures of these antigens were tested with the same pool of 108 serum samples. M1-ELISA: sensitivity 100%, specificity 100%. M2-ELISA: sensitivity 100%, specificity 95%. M3-ELISA: sensitivity 100%, specificity 95%. M4-ELISA: sensitivity 100%, specificity 100%. M4 was characterised by the highest reactivity. To confirm this thesis, a test with a new 128 serum samples was carried out, again sensitivity and specificity were 100%. All serum samples was tested in TLA-ELISA: sensitivity 100%; specificity 100%. Serum samples previously tested with MAT and IFAT.	[84] Poland
M1: SAG1 + MIC1 + MAG1; M2: SAG2 + GRA1 + ROP1; M3: GRA1 + GRA2 + GRA6 Chimeric antigens: MIC1-MAG1-SAG1_S_; SAG1_L_-MIC1-MAG1; SAG2-GRA1-ROP1_S_; SAG2-GRA1-ROP1_L_; GRA1-GRA2-GRA6	*E. coli*	IgG ELISA	IFAT MAT In-house TLA-ELISA	191 serum samples were tested. M1-ELISA: sensitivity 77.9%; specificity 92.2%. M2-ELISA: sensitivity 100%; specificity 100%. M3-ELISA: sensitivity 92.1%; specificity 100%. MIC1-MAG1-SAG1_S_-ELISA: sensitivity 97.9%. SAG1_L_-MIC1-MAG1-ELISA: sensitivity 100%. SAG2-GRA1-ROP1_S_-ELISA: sensitivity 100%. SAG2-GRA1-ROP1_L_-ELISA: sensitivity 100%. GRA1-GRA2-GRA6-ELISA: sensitivity 92.1%. Specificity of all IgG ELISA with recombinant chimeric antigens were 100%. TLA-ELISA: sensitivity 100%; specificity 100%. Serum samples previously tested with MAT and IFAT.	[85] Poland
SAG2	*E. coli*	IgG ELISA	IFAT	60 serum samples were tested.	[86] India
				Compared to IFAT, the SAG2-ELISA had 81.3% sensitivity and 85.7% specificity.	
MAG1	*E. coli*	IgG ELISA	LAT	175 serum samples were tested. 29 (16.6%) serum samples were positive in LAT test. 42 (24%) serum samples positive in MAG1-ELISA. 27 (15.4%) serum samples produced the same results in LAT test and MAG1-ELISA. No additional tests were performed to confirm the presence of anti-*T. gondii* antibodies, so it is difficult to determine the diagnostic utility of MAG1 protein.	[87] Japan
SAG2	*E. coli*	IgG ELISA	-	610 serum samples were tested. 122 (20%) serum samples were positive for the presence of anti-*T. gondii* antibodies. Serum samples were not tested by any commercial assay.	[80] Japan Turkey
GRA7	*E. coli*	IgG ELISA	LAT	111 serum samples were tested.	[88] Egypt
				*T. gondii* infection was detected in 53 (47.8%) serum samples using the LAT test.	
				*T. gondii* infection was detected in 57 (51.4%) serum samples using the GRA7-ELISA.	
				The same result were obtained for both LAT and GRA7 ELISA for 43 (38.7%) serum samples.	
				No additional tests were performed to confirm the presence of anti-*T. gondii* antibodies, so it is difficult to determine the diagnostic utility of GRA7 protein.	
Chimeric antigens: AMA1_N_-SAG2-GRA1-ROP1; AMA1_C_-SAG2-GRA1-ROP1; AMA1-SAG2-GRA1-ROP1; SAG2-GRA1-ROP1-GRA2	*E. coli*	IgG ELISA	IFAT MAT In-house TLA-ELISA	90 serum samples were tested. AMA1_N_-SAG2-GRA1-ROP1-ELISA: sensitivity 97.9%, specificity 97.6%. AMA1_C_-SAG2-GRA1-ROP1-ELISA: sensitivity 95.8%, specificity 95.2%. AMA1-SAG2-GRA1-ROP1-ELISA: sensitivity 97.9%, specificity 100%. SAG2-GRA1-ROP1-GRA2-ELISA: sensitivity 97.9%, specificity 97.6%. TLA-ELISA: sensitivity 97.9%, specificity 100%. Serum samples previously tested with MAT and IFAT.	[89] Poland

Explanation of abbreviations: SA—soluble somatic antigen.

**Table 5:** Recombinant antigens used in detection of anti*-T. gondii* antibodies in bovine serum samples.

Antigen	Expression System	Assay	Reference Test	Results	Reference Country
GRA7	*E. coli*	IgG ELISA	IFA WB In house TLA-ELISA	101 serum samples were tested. GRA7-ELISA identified 28 positive and 73 negative samples. TLA-ELISA identified 22 positive and 79 negative samples. Results were compared with those obtained with IFA and WB, which showed that the GRA7-ELISA is the best method. GRA7-ELISA: sensitivity 96.4%; specificity 98.6%. TLA-ELISA: sensitivity 95.5%; specificity 91.1%.	[79] China
SAG2	*E. coli*	IgG ELISA	IFAT	45 serum samples were tested.	[86] India
				Compared to IFAT, the SAG2-ELISA had 87.1% sensitivity and 85.7% specificity.	
GRA7	*E. coli*	IgG ELISA	-	598 serum samples were tested.	[90] Japan
				44 (7.4%) serum samples were considered positive.	
				No reference tests were performed (neither commercial nor in-house TLA based).	
SAG2	*E. coli*	IgG ELISA	-	377 serum samples were tested.	[80] Japan Turkey
				15 (4%) serum samples were positive for the presence of anti-*T. gondii* antibodies.	
				Serum samples were not tested by any commercial assay.	
GRA7	*E. coli*	IgG ELISA	LAT	301 serum samples were tested.	[88] Egypt
				*T. gondii* infection was detected in 88 (29.2%) serum samples using the LAT test.	
				*T. gondii* infection was detected in 85 (28.2%) serum samples using the GRA7-ELISA.	
				The same result were obtained for both LAT and GRA7 ELISA for 71 (23.6%) serum samples.	
				No additional tests were performed to confirm the presence of anti-*T. gondii* antibodies, so it is difficult to determine the diagnostic utility of GRA7 protein.	
GRA7	*E. coli*	IgG ELISA	-	1438 serum samples were tested.	[91] Japan
				269 (18.7%) serum samples were considered positive.	
				No reference tests were performed (neither commercial nor in-house TLA based).	
SAG2	*E. coli*	IgG ELISA	IFAT	258 serum samples were tested.	[92] India
				115 (44.6%) serum samples give positive results for the presence of anti-*T. gondii* antibodies.	
				Compared to IFAT, the SAG2-ELISA had 80% sensitivity and 88.6% specificity.	

**Table 6:** Recombinant antigens used in detection of anti*-T. gondii* antibodies in caprine serum samples.

Antigen	Expression System	Assay	Reference Test	Results	Reference Country
SAG2	*E. coli*	IgG ELISA	IFAT	63 serum samples were tested.	[86] India
				Compared to IFAT, the SAG2-ELISA had 82.1% sensitivity and 91.4% specificity.	
SAG2	*E. coli*	IgG ELISA	-	249 serum samples were tested.	[80] Japan Turkey
				32 (12.9%) serum samples were positive for the presence of anti-*T. gondii* antibodies.	
				Serum samples were not tested by any commercial assay.	
GRA7	*E. coli*	IgG ELISA	LAT	94 serum samples were tested. *T. gondii* infection was detected in 33 (35.1%) serum samples using the LAT test. *T. gondii* infection was detected in 37 (39.4%) serum samples using the GRA7-ELISA. The same result was obtained for both LAT and GRA7 ELISA for 27 (28.7%) serum samples. No additional tests were performed to confirm the presence of anti-*T. gondii* antibodies, so it is difficult to determine the diagnostic utility of GRA7 protein.	[88] Egypt
SAG1	*E. coli*	IgG ELISA	IFAT	445 serum samples were tested.	[93] India
				189 (42.5%) serum samples were positive for the presence of anti-*T. gondii* antibodies in SAG1-ELISA.	
				Relative to the results obtained in the IFAT, the sensitivity and specificity of the SAG1-ELISA was 92.7% and 90.7%, respectively.	
Chimeric antigens: AMA1_N_-SAG2-GRA1-ROP1; AMA1_C_-SAG2-GRA1-ROP1; AMA1-SAG2-GRA1-ROP1; SAG2-GRA1-ROP1-GRA2	*E. coli*	IgG ELISA	IFAT MAT In-house TLA-ELISA	86 serum samples were tested. AMA1_N_-SAG2-GRA1-ROP1-ELISA: sensitivity 88.9%, specificity 100%. AMA1_C_-SAG2-GRA1-ROP1-ELISA: sensitivity 95.6%, specificity 97.6%. AMA1-SAG2-GRA1-ROP1-ELISA: sensitivity 95.6%, specificity 100%. SAG2-GRA1-ROP1-GRA2-ELISA: sensitivity 57.8%, specificity 95.1%. TLA-ELISA: sensitivity 97.8%, specificity 100%. Serum samples were previously tested with MAT and IFAT.	[89] Poland

**Table 7:** Recombinant antigens used in detection of anti*-T. gondii* antibodies in porcine serum samples.

Antigen	Expression System	Assay	Reference Test	Results	Reference Country
H4; H11	*E. coli*	IgG ELISA	MAT In-house NAE-ELISA	Recombinant antigens are better recognised by specific antibodies contained in some samples of sera from pigs up to 9 weeks after infection with *T. gondii.*	[94] USA
				Recombinant antigens are generally less reactive than the native antigen extract, in particular, the reactivity with sera takes longer to develop after *T. gondii* infection.	
B427 (MAG1); C55 (GRA2); V22 (SAG2); MBP30 (SAG1); C51	*E. coli*	IgG ELISA	In-house NTA-ELISA	Antigens with the best properties are C51, V22 and MBP30 based on the difference between the values obtained for negative and positive sera.	[95] USA
				C51 and MBP30 antigens were correctly recognised by specific antibodies in sera of pigs infected with *T. gondii* 52 weeks after infection.	
MIC3; SAG1	*E. coli*	IgG LAT IgG ELISA	-	MIC3-LAT test correctly identified all 50 seropositive sera from experimentally infected pigs; the test specificity was 100%.	[96] China
				SAG1-ELISA test correctly identified 48 seropositive sera; the test specificity was 100%.	
				Examining 256 undefined samples of pig sera using the MIC3-LAT test were identified 103 as positive; specific anti-*T. gondii* antibodies were detected in 94 sera in the SAG1-ELISA.	
				Positive agreement between MIC3-LAT and SAG1-ELISA was 91.3% for undefined serum samples, and 92.8% for all sera used.	
MAG1; SAG1; GRA7; M1: MAG1 + SAG1; M2: MAG1 + GRA7; M3: SAG1 + GRA7; M4: MAG1 + SAG1 + GRA7	*E. coli*	IgG ELISA	MAT In-house TLA-ELISA	120 serum samples were tested.	[97] Poland
				MAG1-ELISA: sensitivity 64%. SAG1-ELISA: sensitivity 85.3%.	
				GRA7-ELISA: sensitivity 81.3%.	
				M1-ELISA: sensitivity 86.7%.	
				M2-ELISA: sensitivity 89.2%.	
				M3-ELISA: sensitivity 92%.	
				M4-ELISA: sensitivity 97.3%.	
				Specificity of all IgG ELISA with recombinant antigens was 100%.	
				TLA-ELISA: sensitivity 100%; specificity 100%.	
				Serum samples were previously tested with MAT and IFAT.	
SAG2; GRA7; GRA14	*E. coli*	IgG ELISA IgM ELISA IgG ICT	LAT	From based on recombinant antigens, the GRA7 antigen is best recognised by anti-*T. gondii* antibodies.	[98] Japan
				Results of the commercial LAT test indicated that the sensitivity of the GRA7-ELISA was 90.6%, the specificity was 85.2%, and the compliance was 88.1%.	
				Results of the commercial LAT test, indicated that the sensitivity of the GRA7-ICT test was 71.9%, the specificity was 96.3%, and the compliance was 83.1%.	
M1: SAG1 + MIC1 + MAG1; M2: SAG2 + GRA1 + ROP1; M3: GRA1 + GRA2 + GRA6; Chimeric antigens: MIC1-MAG1-SAG1_S_; SAG1_L_-MIC1-MAG1; SAG2-GRA1-ROP1_S_; SAG2-GRA1-ROP1_L_; GRA1-GRA2-GRA6	*E. coli*	IgG ELISA	IFAT MAT In-house TLA-ELISA	168 serum samples were tested. M1-ELISA: sensitivity 88.9%; specificity 100%. M2-ELISA: sensitivity 81.5%; specificity 100%. M3-ELISA: sensitivity 54.3%; specificity 100%. MIC1-MAG1-SAG1_S_-ELISA: sensitivity 45.7%. SAG1_L_-MIC1-MAG1-ELISA: sensitivity 90.1%. SAG2-GRA1-ROP1_S_-ELISA: sensitivity 28.4%. SAG2-GRA1-ROP1_L_-ELISA: sensitivity 93.8%. GRA1-GRA2-GRA6-ELISA: sensitivity 96.3%. Specificity of all IgG ELISA with recombinant chimeric antigens was 100%. TLA-ELISA: sensitivity 100%; specificity 100%. Serum samples previously tested with MAT and IFAT.	[85] Poland
GRA7	*E. coli*	IgG ELISA	-	205 serum samples were tested.	[90] Japan
				30 (14.6%) serum samples were considered positive.	
				No reference tests were performed (neither commercial nor in-house TLA based).	
CCp5A; OWP1	*E. coli*	IgG WB IgG ELISA	WB In-house STAg-ELISA	44 serum samples were tested.	[99] Brazil
				CCp5A-WB: detection of infection.	
				OWP1-WB: no detection of infection.	
				STAg-WB: detection of infection.	
				CCp5A-ELISA: sensitivity 100%, specificity undefined.	
				STAg-ELISA: sensitivity 100%, specificity undefined.	
				No additional tests were performed to confirm the presence of anti-*T. gondii* antibodies, so it is difficult to determine the diagnostic utility of recombinant proteins.	
GRA7; OWP8; CCp5A	*E. coli*	IgG ELISA	-	90 serum samples were tested.	[100] China
				GRA7-ELISA: sensitivity 16.7%, specificity undefined.	
				OWP8-ELISA: sensitivity 12,2%, specificity undefined.	
				CCp5A-ELISA: sensitivity 12.2%, specificity undefined.	
				No tests were performed to confirm the presence of anti-*T. gondii* antibodies, so it is difficult to determine the diagnostic utility of recombinant proteins.	

Explanation of abbreviations: NAE—native antigen extract, NTA—native *T. gondii* antigen, STAg—soluble *Toxoplasma* antigen.

**Table 8:** Recombinant antigens used in detection of anti*-T. gondii* antibodies in equine serum samples.

Antigen	Expression System	Assay	Reference Test	Results	Reference Country
M1: SAG1 + MIC1 + MAG1; M2: SAG2 + GRA1 + ROP1; M3: GRA1 + GRA2 + GRA6; Chimeric antigens: MIC1-MAG1-SAG1_S_; SAG1_L_-MIC1-MAG1; SAG2-GRA1-ROP1_S_; SAG2-GRA1-ROP1_L_; GRA1-GRA2-GRA6	*E. coli*	IgG ELISA	IFAT MAT In-house TLA-ELISA	86 serum samples were tested. M1-ELISA: sensitivity 88.9%; specificity 100%. M2-ELISA: sensitivity 77.8%; specificity 100%. M3-ELISA: sensitivity 66.7%; specificity 100%. MIC1-MAG1-SAG1_S_-ELISA: sensitivity 75%. SAG1_L_-MIC1-MAG1-ELISA: sensitivity 77.8%. SAG2-GRA1-ROP1_S_-ELISA: sensitivity 50%. SAG2-GRA1-ROP1_L_-ELISA: sensitivity 100%. GRA1-GRA2-GRA6-ELISA: sensitivity 86.1%. Specificity of all IgG ELISA with recombinant chimeric antigens was 100%. TLA-ELISA: sensitivity 100%; specificity 100%.	[85] Poland
SAG2	*E. coli*	IgG ELISA	-	616 serum samples were tested. 285 (46.3%) serum samples were positive for the presence of anti-*T. gondii* antibodies. Serum samples were not tested by any commercial assay.	[80] Japan Turkey

**Table 9:** Recombinant antigens used in detection of anti*-T. gondii* antibodies in chicken serum samples.

Antigen	Expression System	Assay	Reference Test	Results	Reference Country
CCp5A; OWP1	*E. coli*	IgY ELISA	WB In-house STAg-ELISA	113 serum samples were tested.	[99] Brazil
				CCp5A-ELISA: sensitivity 70%, specificity undefined.	
				OWP1-ELISA: sensitivity 74% specificity undefined.	
				STAg-ELISA: sensitivity 80%, specificity undefined.	
				No tests were performed to confirm the presence of anti-*T. gondii* antibodies, so it is difficult to determine the diagnostic utility of recombinant proteins.	
GRA1; GRA7	*E. coli*	IgY ELISA	In-house TSA-ELISA	110 serum samples were tested.	[101] China
				GRA1-ELISA: sensitivity 81.3%, specificity 94.7%.	
				GRA7-ELISA: sensitivity 100%, specificity 98.9%.	
				TSA-ELISA: sensitivity 93.8%, specificity 97.9%.	
				Serum samples were previously tested in WB to confirm the presence of anti-*T. gondii* antibodies.	
SAG2	*E. coli*	IgY ELISA	Histopathology IHC	304 serum samples were tested.	[102] Egypt
				SAG2-ELISA: sensitivity 11.2%, specificity undefined.	
				6.9% cases confirmed by other testes—histopathology and IHC.	
				Test compliance was 95.7%.	
GRA7; OWP8; CCp5A	*E. coli*	IgY ELISA	-	96 serum samples were tested.	[100] China
				GRA7-ELISA: Sensitivity 10.4%, specificity undefined.	
				OWP8-ELISA: 13,5%, specificity undefined.	
				CCp5A-ELISA: 9.4%, specificity undefined.	
				No tests were performed to confirm the presence of anti-*T. gondii* antibodies, so it is difficult to determine the diagnostic utility of recombinant proteins.	
GRA8	*E. coli*	IgY LACA IgY ELISA	-	86 serum samples were tested (21 from experimentally infected chicken and 65 from chicken without anti-*T. gondii* antibodies). Purified Nluc-GRA8-LACA: sensitivity 90.5%, specificity 95.4%. Unpurified Nluc-GRA8-LACA: sensitivity 85.7%, specificity 96.9%. GRA8-ELISA: sensitivity 85.7%, specificity 84.6%%. Screening Nluc-GRA8-LACA was performed using 267 sera from free-range chicken and 100 sera from closed-house broiler chicken. Presence of anti-*T. gondii* antibodies was found in 10.9% of free-range chickens.	[103] Japan

Explanation of abbreviations: Immunoglobulin Y (IgY) in birds corresponds to IgG in mammals, TSA—*Toxoplasma* soluble antigens, IHC—immunohistochemistry, LACA—luciferase-linked antibody capture assay.

**Table 10:** Recombinant antigens used in detection of anti*-T. gondii* antibodies in donkey serum samples.

Antigen	Expression System	Assay	Reference Test	Results	Reference Country
GRA7	*E. coli*	IgG ELISA	LAT	146 serum samples were tested. *T. gondii* infection was detected in 39 (26.7%) serum samples using the LAT test. *T. gondii* infection was detected in 42 (28.8%) serum samples using the GRA7-ELISA. The same result were obtained for both LAT and GRA7 ELISA for 27 (22.6%) serum samples. No additional tests were performed to confirm the presence of anti-*T. gondii* antibodies, so it is difficult to determine the diagnostic utility of GRA7 protein.	[88] Egypt
